# Mumps Epidemiology in the Autonomous Province of Vojvodina, Serbia: Long-Term Trends, Immunization Gaps, and Conditions Favoring Future Outbreaks

**DOI:** 10.3390/vaccines13080839

**Published:** 2025-08-06

**Authors:** Mioljub Ristić, Vladimir Vuković, Smiljana Rajčević, Marko Koprivica, Nikica Agbaba, Vladimir Petrović

**Affiliations:** 1 Institute of Public Health of Vojvodina, 21000 Novi Sad, Serbia; smiljana.rajcevic@mf.uns.ac.rs (S.R.); marko.koprivica@izjzv.org.rs (M.K.); nikica.agbaba@izjzv.org.rs (N.A.); vladimir.petrovic@mf.uns.ac.rs (V.P.); 2 Department of Epidemiology, Faculty of Medicine, University of Novi Sad, 21000 Novi Sad, Serbia

**Keywords:** mumps, vaccination coverage, incidence, complications, epidemiology, surveillance, infectious disease, epidemics, Serbia, AP Vojvodina

## Abstract

**Background/Objectives**: Mumps remains a relevant vaccine-preventable disease globally, especially in settings where immunization coverage fluctuates or vaccine-induced immunity wanes. This study aimed to assess long-term trends in mumps incidence, vaccination coverage, clinical outcomes, and demographic characteristics in the Autonomous Province of Vojvodina (AP Vojvodina), Serbia, over a 47-year period. **Methods**: We conducted a retrospective observational study using surveillance data from the Institute of Public Health of Vojvodina. Analyses included annual mumps incidence rates (1978–2024), coverage with mumps-containing vaccines (MuCVs; 1986–2024), monthly case counts, and individual-level case data for the 1997–2024 period. Variables analyzed included age, month of notification, gender, vaccination status, presence of clinical complications, and the method used for case confirmation. **Results**: Following the introduction of MuCV in 1986, the mumps incidence markedly declined, with limited resurgences in 2000, 2009, and 2012. Between 1997 and 2024, a total of 1358 cases were reported, with 62.7% occurring in males. Over time, the age distribution shifted, with adolescents and young adults being increasingly affected during the later (2011–2024) observed period. In 2012, the highest age-specific incidence was observed among individuals aged 10–19 and 20–39 years (49.1 and 45.5 per 100,000, respectively). Vaccination coverage for both MuCV doses was suboptimal in several years. The proportion of unvaccinated cases decreased over time, while the proportion with unknown vaccination status increased. Mumps-related complications—such as orchitis, pancreatitis, and meningitis—were rare and predominantly affected unvaccinated individuals: 84.2% of orchitis, 40.0% of pancreatitis, and all meningitis cases. Only two pancreatitis cases (40.0%) were reported after one MMR dose, while fully vaccinated individuals (two doses) had one orchitis case (5.3%) and no other complications. Laboratory confirmation was applied more consistently from 2009 onward, with 49.6% of cases confirmed that year (58 out of 117), and, in several years after 2020, only laboratory-confirmed cases were reported, indicating improved diagnostic capacity. **Conclusions**: Despite substantial progress in controlling mumps, gaps in vaccine coverage, waning immunity, and incomplete vaccination records continue to pose a risk for mumps transmission. Strengthening routine immunization, ensuring high two-dose MuCV coverage, improving vaccination record keeping, and enhancing laboratory-based case confirmation are critical. Consideration should be given to booster doses in high-risk populations and to conducting a seroepidemiological study to estimate the susceptible population for mumps in AP Vojvodina.

## 1. Introduction

Mumps is an acute viral illness caused by the mumps virus, a member of the Paramyxoviridae family, characterized by unilateral or bilateral tenderness or inflammatory swelling of the parotid glands and, in some cases, complications such as orchitis, pancreatitis, and meningitis [1,2]. In unvaccinated children, mumps infection is asymptomatic in 20–40% of cases [1].

Despite being endemic worldwide, mumps is not a notifiable disease in many countries, which results in scarce or entirely absent routine surveillance data [1,3].

Although the disease has been largely controlled in many countries through widespread vaccination, mumps continues to cause outbreaks, especially in populations with suboptimal vaccine coverage or due to waning vaccine-induced immunity [1,4,5].

The introduction of the mumps-containing vaccine (MuCV) has led to a dramatic decline in incidence globally [1,2]. However, despite high two-dose measles–mumps–rubella (MMR) vaccine coverage, periodic resurgences have been documented in Europe and North America, often affecting adolescents and young adults [6,7,8]. These outbreaks raise concerns about the duration of vaccine-induced immunity, vaccine effectiveness, and potential gaps in immunization programs [8,9,10].

In Serbia, mumps became a nationally notifiable disease in 1976. In the Autonomous Province of Vojvodina (AP Vojvodina)—the northern province of Serbia, accounting for approximately 26% of the national population (1,740,230 inhabitants according to the 2022 Census)—systematic mumps surveillance data have been available since 1978 onward. Mandatory mumps immunization for children and contacts during outbreaks throughout the entire territory of Serbia was introduced in 1986 with the administration of the combined measles–mumps (MM) vaccine, targeting children aged 12–15 months. In 1993, the MM vaccine was replaced by the combined measles–mumps–rubella (MMR) vaccine. A two-dose MMR vaccination schedule was implemented in 1994, with doses administered at 12–15 months and at 12 years of age. In 2006, the timing of the second dose was revised to be given at preschool age (6–7 years) [11]. In Serbia, the first mumps vaccine (MM), introduced into the childhood immunization schedule in 1986, contained the L-Zagreb strain and was produced by the Institute of Immunology in Zagreb. Since 1993, the MMR vaccine containing the Urabe AM9 strain—primarily the TRIMOVAX MÉRIEUX vaccine manufactured by Sanofi Pasteur—has been in use. Exceptions were the years 2001–2002 and the period from 2015 to 2017, when the Jeryl Lynn strain was used in the GlaxoSmithKline MMR vaccine, and the period from 2018 to 2024, when the Jeryl Lynn strain was used in the Merck Sharp & Dohme MMR vaccine [11,12]. According to estimates, approximately 16,000 children in the territory of AP Vojvodina require initiation of mandatory mumps immunization each year [11].

This study provides a comprehensive analysis of mumps epidemiology in AP Vojvodina, focusing on long-term incidence trends, vaccination coverage against mumps, the demographic and clinical characteristics of reported cases, and the distribution of mumps-related complications. The findings aim to inform public health strategies for the prevention and control of mumps in comparable settings.

## 2. Materials and Methods

### 2.1. Study Design and Data Sources

This retrospective observational study analyzed all officially reported mumps cases in AP Vojvodina. It aimed to assess the crude mumps incidence (from 1978 to 2024) and MuCV coverage (from 1986 to 2024). For the period 1997–2024, when only the MMR vaccine was available [11], the dataset included the number of mumps cases and the coverage rates for both the first (MMR I) and second (MMR II) vaccine doses by age group in AP Vojvodina.

For each mumps case, we collected the following variables: age, month of notification, gender, vaccination status, presence of clinical complications, and the method used for case confirmation. As previously described [12], mumps surveillance in Serbia has been conducted in accordance with the World Health Organization (WHO) criteria. A clinical case of mumps is defined as an individual presenting with acute onset of unilateral or bilateral, tender, self-limited swelling of the parotid or other salivary glands, lasting for two or more days and without another apparent cause. A laboratory-confirmed case is defined as a clinical case of mumps confirmed through serological and/or molecular methods. An epidemiologically linked case meets the clinical case definition and has an established epidemiological connection to a laboratory-confirmed case [13].

Mumps-specific incidence rates, gender distribution, and clinical presentations were stratified and analyzed within the following six age groups: <1 year, 1–4 years, 5–9 years, 10–19 years, 20–39 years, and ≥40 years.

### 2.2. Data Collection and Variables

The following variables were included in the analysis.

- Annual mumps incidence rate: Number of reported cases per 100,000 total population per year in AP Vojvodina from 1978 to 2024.
- Age-specific mumps incidence rate: Number of reported cases per 100,000 population within specific age groups in AP Vojvodina from 1997 to 2024.
- MuCV I (MMR I) coverage: Annual percentage of the target population (at 12–15 months) receiving the first dose of MuCV from 1986 to 2024.
- MuCV II (MMR II) coverage: Annual percentage of the target population (at 12 years in the period 1994–2005; at 6–7 years in the period 2006–2024) receiving the second dose of MuCV from 1994 to 2024.

In Serbia, vaccination coverage is assessed using the administrative method, which calculates the proportion of vaccine doses administered to the target birth cohorts from the previous year [11,14,15].

Individuals without any documented mumps vaccination were classified as “unvaccinated”. During the period 1997–2024, individuals with missing or indeterminate immunization records were categorized as having an “unknown” mumps vaccination status. Those with documentation of one or two administered doses of the MMR vaccine were classified as having received MMR I or MMR II, respectively.

### 2.3. Data Analysis

Descriptive statistical methods were used to assess trends in mumps incidence (1978–2024) and vaccination coverage throughout the 1986–2024 period. Time-series graphs were constructed to visualize trends, fluctuations, and possible outbreak patterns. As previously described [15], all annual data were officially reported to the Center for Disease Control and Prevention at the Institute of Public Health of Vojvodina (IPHV), Novi Sad, as part of routine communicable disease surveillance.

### 2.4. Ethical Considerations

Consistent with previously applied methodologies [15,16], and in accordance with national regulations and institutional guidelines in Serbia, retrospective analyses based on anonymized data are exempt from ethical committee approval. The authors were not involved in the clinical management of the included patients and had no access to personally identifiable information at any stage of data collection or analysis.

## 3. Results

### 3.1. Trends in Mumps Incidence and Vaccination Coverage

**Figure 1:**
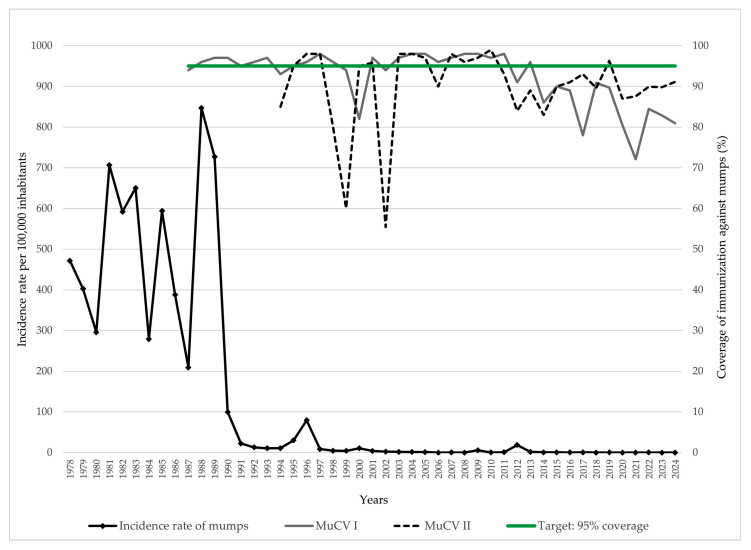
Trends of mumps incidence and MuCV vaccination coverage in AP Vojvodina, 1978–2024. Legend: MuCV—mumps-containing vaccine.

The incidence rate of mumps in AP Vojvodina demonstrated significant fluctuations over the 47-year observation period. During the pre-vaccination era (1978–1986), the incidence remained high, ranging from 209.1 to 706.4 cases per 100,000 inhabitants. Following the introduction of the first dose of MuCV in 1986 and subsequent improvements in coverage, a marked decline in incidence was observed, despite temporary resurgences in 1988, 1989, and 1996 (846.7, 727.0, and 79.4 per 100,000, respectively). Since 2000, the incidence has remained exceptionally low, with most years recording fewer than five cases per 100,000 inhabitants. Intermittent resurgences included 2009 (5.7 per 100,000) and 2012 (18.6 per 100,000), likely reflecting localized outbreaks. Between 2014 and 2024, the incidence remained consistently low, at or below 0.5 per 100,000 annually. Vaccination coverage with MuCV I remained below the target of 95% in several years, with the lowest coverage reported in 2000 (82.0%), 2017 (78.0%), and 2021 (72.1%). Similarly, coverage with MuCV II fell short of the 95% target in multiple years. The lowest coverage rates for MuCV II were observed in 1999 (60.0%), 2002 (55.4%), and 2014 (83.0%) (Figure 1).

### 3.2. Age-Specific Distribution of Mumps Across Two Distinct Periods

**Figure 2:**
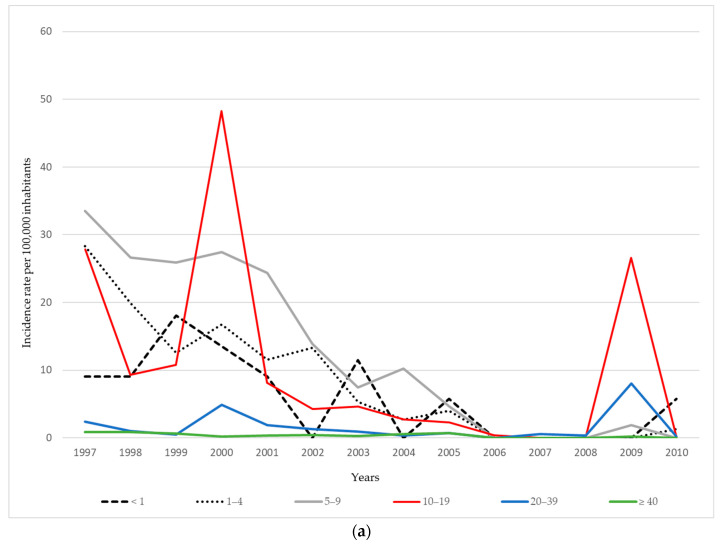

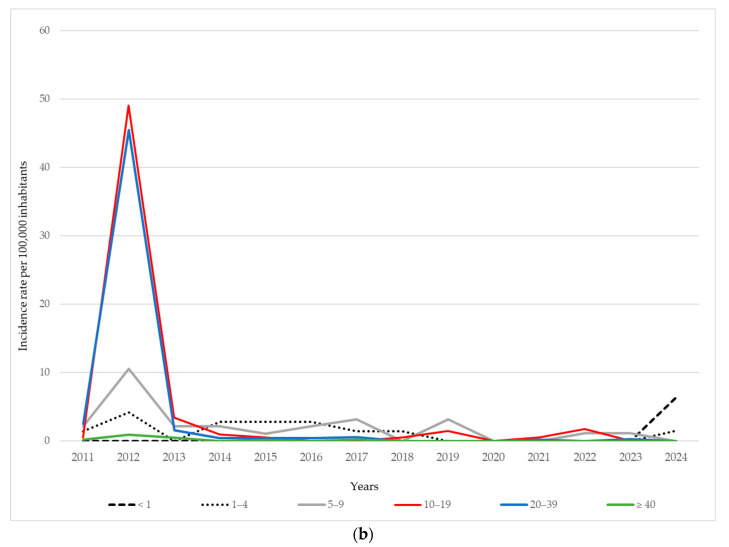
Age-specific incidence of mumps per 100,000 inhabitants in AP Vojvodina: (**a**) during 1997–2010; (**b**) during 2011–2024.

During the late 1990s, the highest mumps incidence rates in AP Vojvodina were consistently recorded among children aged 5–9 and adolescents aged 10–19 years. In 1997, incidence peaked at 33.5 per 100,000 population in the 5–9 age group and 27.9 per 100,000 in the 10–19 age group, whereas adults aged ≥ 20 years continuously exhibited the lowest incidence rates during the observed period. During the 2000 outbreak, the highest burden was observed among adolescents aged 10–19 years (48.3 per 100,000), followed by children aged 5–9 years (27.4 per 100,000). After 2000, the incidence rates markedly declined across all age groups, with occasional resurgences, such as in 2009, when an increase was again registered among adolescents aged 10–19 years (26.6 per 100,000), as well as among young adults aged 20–39 years (8.1 per 100,000) (Figure 2a).

During the 2011–2024 period, the overall incidence of mumps in AP Vojvodina remained relatively low compared to the previous period, with sporadic increases observed in certain age groups and years. A moderate resurgence occurred in 2012, with the highest incidence observed among adolescents aged 10–19 years (49.1 per 100,000) and young adults aged 20–39 years (45.5 per 100,000), while school-aged children at 5–9 years also showed elevated rates (10.6 per 100,000). In subsequent years, the incidence declined across all age groups, with only minor fluctuations. The most affected age groups during the post-2012 period were typically children aged 5–9 years and adolescents, although the rates remained below 3.5 per 100,000 in most years. Interestingly, in 2024, a small increase in incidence was recorded among infants (<1 year: 6.4 per 100,000), a group not eligible for MMR vaccination, and among children aged 1–4 years (1.5 per 100,000), while no cases were registered in older age groups. From 2020 to 2023, the incidence remained extremely low or zero in all age categories, which may partly reflect reduced virus circulation during and after the COVID-19 pandemic and associated public health measures (Figure 2b).

### 3.3. Monthly Distribution of Reported Mumps Cases, 1997–2024

**Figure 3:**
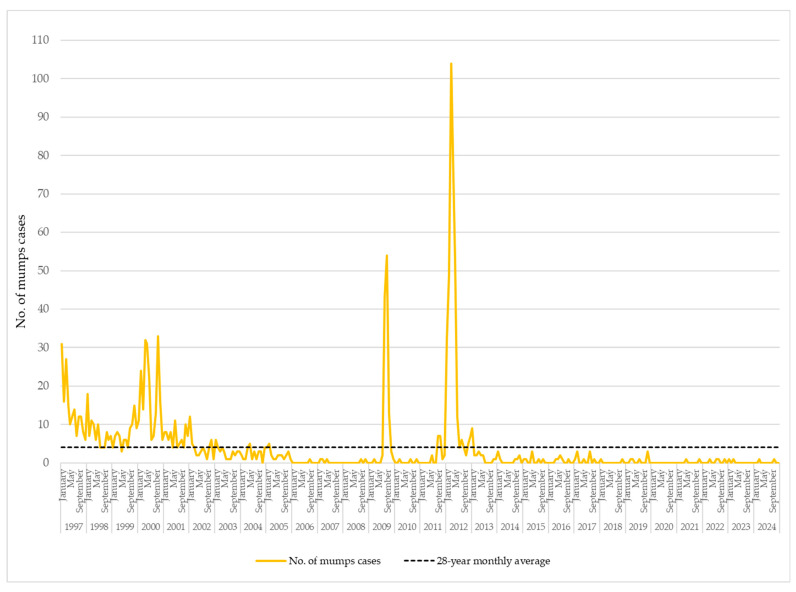
Monthly distribution of mumps cases in AP Vojvodina, 1997–2024.

Over the 28-year period from January 1997 to December 2024, the monthly number of reported mumps cases in AP Vojvodina exhibited substantial fluctuations, indicative of both sustained endemic transmission and intermittent outbreak activity. The calculated long-term monthly average was 4.04 cases. Notably, during the early years of surveillance—particularly from 1997 to 2005—57.7% of the total 1358 mumps cases recorded over the entire 1997–2024 period occurred within this nine-year span. In 1997, notifications peaked at 31 cases in January and 27 in March. Similarly, in 2000, a marked increase was observed, with 32 cases in April, 31 in May, and 33 in October, pointing to a likely localized outbreak. This intermittent pattern of increased transmission persisted through 2001 and 2002, with several monthly peaks interspersed with periods of moderate activity. Following 2005, a downward trend in monthly case counts became apparent, with many months—and, in some years, entire calendar years—reporting only sporadic or no cases. Nonetheless, a resurgence occurred in 2009, with 117 reported cases, signaling a short outbreak. This was followed by a large-scale epidemic in 2012, when 359 cases were reported. During this epidemic, the monthly case numbers greatly surpassed the long-term average, particularly in March (104 cases), April (81), and May (53). This represented the most intense period of mumps transmission in nearly three decades. From 2013 onwards, the disease was reported only sporadically, with the majority of months registering few or no cases (Figure 3).

### 3.4. Distribution of Clinically or Epidemiologically Linked and Laboratory-Confirmed Mumps Cases

**Figure 4:**
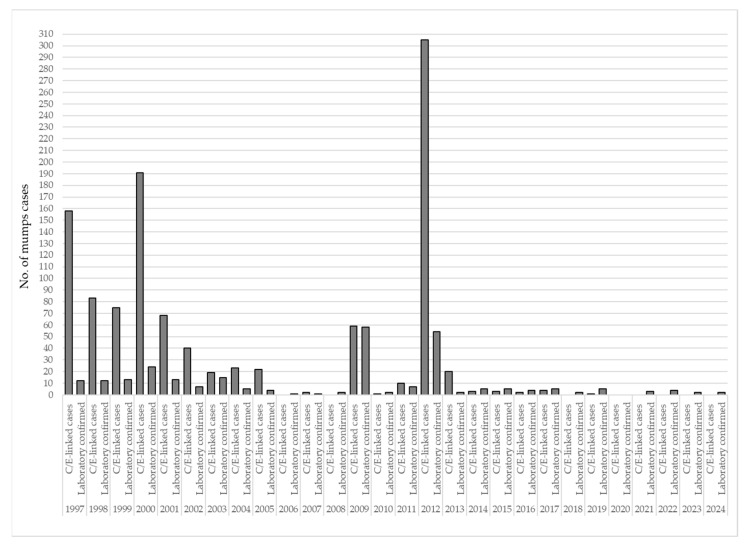
Distribution of clinically or epidemiologically linked and laboratory-confirmed mumps cases in AP Vojvodina, 1997–2024. Legend: C/E-linked cases—clinically or epidemiologically linked mumps cases.

Between 1997 and 2024, 1089 (80.2%), mumps cases were clinically or epidemiologically linked (C/E-linked), while 269 (19.8%) were laboratory-confirmed mumps cases. C/E-linked cases predominated in the earlier years of surveillance, particularly during outbreaks in 1997 (*n* = 158) and 2000 (*n* = 191), but also in 2012 (*n* = 305). In contrast, laboratory confirmation was more consistently applied in the later years, particularly from 2009 onward, when the proportion of confirmed cases increased. For example, in 2009, 58 out of 117 cases (49.6%) were laboratory-confirmed. In several years after 2020, only laboratory-confirmed cases were reported (Figure 4).

### 3.5. Vaccination Status of Mumps Cases in Two Distinct Periods

Between 1997 and 2010, the majority of reported mumps cases in AP Vojvodina occurred among unvaccinated individuals (57.5%), followed by those who had received a single dose of the MMR vaccine (24.4%). Only 15.8% of cases were reported among individuals who had received two doses of the MMR vaccine, while the vaccination status was unknown for 2.3% of cases. In the subsequent period (2011–2024), the proportion of unvaccinated cases decreased to 45.3%, and the share of cases among individuals with one MMR dose dropped to 5.8%. Similarly, the proportion of cases in individuals who had received two MMR doses declined to 8.7%. However, the proportion of cases with an unknown vaccination status increased to 40.2%.

**Figure 5:**
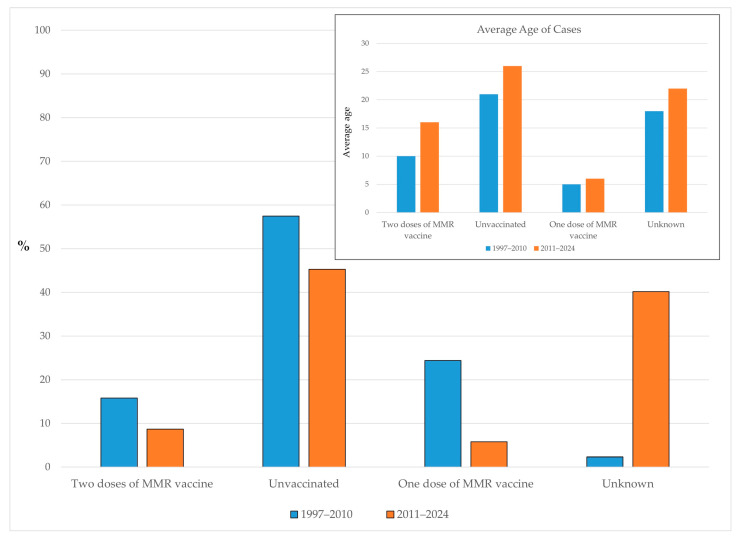
Proportion of mumps cases by vaccination status and average age in AP Vojvodina across two distinct periods.

Among individuals who had received two doses of the MMR vaccine, the mean age increased from 10 years in the period of 1997–2010 to 16 years in 2011–2024. A similar upward shift was observed among unvaccinated cases, with the mean age rising from 21 to 26 years. For cases who had received only one dose of the MMR vaccine, the mean age remained low, increasing slightly from 5 to 6 years. Among individuals with an unknown vaccination status, the mean age also rose, from 18 years in the earlier period to 22 years in the latter (Figure 5).

### 3.6. Distribution of Mumps by Gender

**Figure 6:**
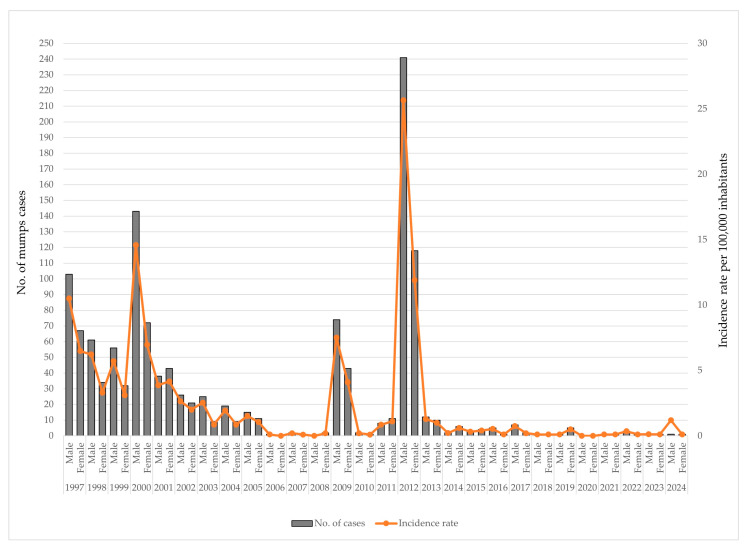
Annual number of mumps cases and incidence rates by gender in AP Vojvodina, 1997–2024.

Throughout the study period, mumps consistently demonstrated a male predominance, with higher notification rates observed in nearly every year. During the early surveillance period (1997–2001), the incidence rates were relatively high in both sexes, particularly in 2000, when the incidence among males peaked at 14.6 per 100,000 population and among females at 7.0 per 100,000. A declining trend followed from 2002 to 2008, with incidence rates falling below 3 per 100,000 for both sexes. A temporary resurgence was observed in 2009, with the rates increasing to 7.5 per 100,000 among males and 4.1 among females, preceding the most substantial outbreak of the period, which occurred in 2012. In that year, the incidence rate reached 25.6 per 100,000 in males—more than double the rate in females (11.9 per 100,000). From 2013 onwards, the mumps incidence declined sharply and remained at low endemic levels, with annual rates rarely exceeding 1 per 100,000 in either gender of patients (Figure 6).

**Figure 7:**
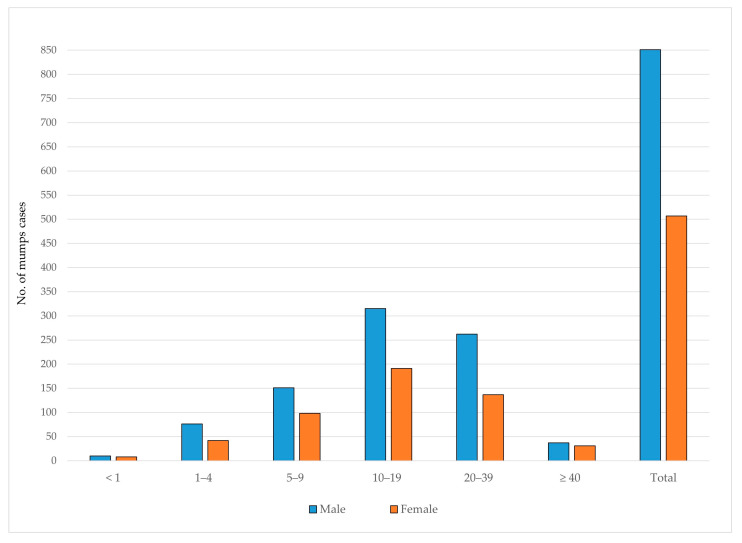
Age and gender distribution of reported mumps cases in AP Vojvodina, 1997–2024.

Between 1997 and 2024, 851 (62.7%) of mumps cases occurred among males and 507 (37.3%) among females. In all age groups, the number of male cases exceeded that of females. The highest number of mumps cases was observed in the 10–19-year age group for both genders, accounting for 37.0% of male and 37.7% of female cases (Figure 7).

The distribution of mumps cases by age group and gender over the entire study period (1997–2024) is provided in the Supplementary Materials (Figure S1a–f).

### 3.7. Distribution of Uncomplicated Mumps Cases and Mumps-Related Complications

Among 1358 reported mumps cases with known clinical outcomes, the majority (1326; 97.6%) were classified as uncomplicated. The majority of uncomplicated mumps cases (67.5%) occurred during the period 1997–2010, while most complications—including orchitis (57.9%) and all cases of pancreatitis (100%)—were recorded in the later period, 2011–2024. Mumps meningitis was predominantly observed in the earlier period (87.5%).

Orchitis was reported in 19 patients, with the vast majority (94.7%) aged 20–39 years. Pancreatitis was reported in five patients, mostly aged 5–19 years. Meningitis occurred in eight patients, with the highest proportion (62.5%) among those aged 10–19 years.

Regarding vaccination status, the highest proportion of all complications occurred among unvaccinated individuals, accounting for 84.2% of orchitis cases, 40.0% of pancreatitis cases, and all meningitis cases (100%). No complications were observed among individuals who had received one dose of the MMR vaccine, except for two pancreatitis cases (40.0%). Among fully vaccinated individuals (two doses), only one case of orchitis (5.3%) and no other complications were recorded (Table 1).

## 4. Discussion

This study provides a comprehensive overview of mumps epidemiology in AP Vojvodina. The findings demonstrate notable changes in disease incidence, age-specific distribution, and vaccination coverage, along with important shifts in clinical presentation and surveillance approaches.

### 4.1. Long-Term Incidence Trends and Vaccination Coverage with Focus on Regional Mumps Epidemiology

To support the goal of eliminating measles and rubella globally by 2030, the Measles & Rubella Initiative developed the Measles and Rubella Strategic Framework 2021–2030, under the broader umbrella of the Immunization Agenda 2030 [17]. In line with the WHO objective to achieve the high-level control of mumps as well, 121 countries have adopted combined live attenuated vaccines against measles, mumps, and rubella (MMR) in their national immunization programs [10].

The introduction of MuCV in 1986 and the subsequent implementation of a two-dose MMR schedule led to a marked decline in mumps incidence across AP Vojvodina. This mirrors trends observed in other countries that have successfully implemented routine MMR immunization [1,10,18,19]. In AP Vojvodina, mumps has not been fully eliminated, despite a previously favorable epidemiological situation. Outbreaks were recorded in 2000, 2009, and, most notably, in 2012, although the incidence rates during these events were considerably lower compared to the pre-vaccine era. These outbreaks coincided with periods of suboptimal vaccination coverage. Fluctuations in annual MMR coverage in AP Vojvodina—particularly for MMR I in 2000 (82%), 2017 (78%), and 2021 (72%) and for MMR II in 1998 (80%), 1999 (60%), 2002 (55%), and 2014 (83%)—highlight that even short-term declines in immunization rates can result in cohorts remaining susceptible to mumps virus transmission. A similar epidemiological pattern of mumps has been observed across the European Union/European Economic Area (EU/EEA). In 2022, the annual incidence rate in the EU/EEA was 0.7 per 100,000 population [20], which is comparable to the rate recorded in AP Vojvodina (0.2 per 100,000). Similar declines in MMR vaccination coverage have been documented not only in our region but also in several neighboring countries of Serbia, reflecting a broader regional challenge [15,21].

In the period between 2000 and 2023, several large-scale mumps outbreaks were reported in Serbia’s neighboring countries. Notably, Romania experienced a prolonged epidemic between 2000 and 2005, with the peak incidence in 2004, exceeding 3000 cases per million inhabitants. Albania also recorded an elevated mumps incidence from 2000 to 2005, with a peak in 2002, nearing 1000 cases per million. North Macedonia reported a major outbreak in 2008–2009, with the incidence surpassing 5000 cases per million in 2009. Bulgaria registered an incidence of 741 per million in 2008, while Bosnia and Herzegovina experienced significant outbreaks in 2011 and 2012, with incidence rates exceeding 2000 per million in both years. In contrast, Serbia and its other neighboring countries—Croatia, Hungary, and Montenegro—recorded relatively low mumps incidences during the same period, not exceeding 164.2 cases per million, with the highest rate observed in Montenegro in 2012 [21]. That same year, an outbreak also occurred in AP Vojvodina, Serbia, with several cases epidemiologically linked to importation from neighboring Bosnia and Herzegovina. More specifically, the imported cases involved students from the University of Novi Sad who had spent the Christmas and New Year holidays in Bosnia and Herzegovina [12,22]. Otherwise, the 2011–2012 outbreak in Bosnia and Herzegovina represented the largest mumps epidemic in the country since the introduction of the MMR vaccine in 1980 [23].

### 4.2. Age-Specific Trends and Epidemiological Shifts

The age distribution of mumps cases has changed markedly over time. In earlier years, the highest incidence was observed among children aged 5–9 and 10–19 years. However, during more recent outbreaks—particularly the one in 2012—adolescents (10–19 years) and young adults (20–39 years) emerged as the most affected age groups. This shift likely reflects a combination of factors, including, foremost, an inadequate level of immunization coverage (below 95%) for both the first and second doses of the MMR vaccine—especially between 2012 and 2024—incomplete protection conferred by vaccination, waning immunity among individuals vaccinated in early childhood, gaps in vaccine uptake during the initial years of the immunization program, and the high intensity of social contact within these age groups, which facilitates virus transmission [1,4,5,6,7,8,9,10,18,20]. Of particular concern is the increased incidence of mumps among infants in AP Vojvodina in 2024—a population not yet eligible for MMR vaccination. This finding underscores the critical importance of achieving and maintaining herd immunity through high vaccination coverage in the general population to safeguard vulnerable groups, especially those who cannot yet be vaccinated [1,20]. Consistent with the findings from our study based on routine mumps surveillance data—and in the absence of seroprofile data on immunity to the mumps virus in the general population of AP Vojvodina—recently published seroepidemiological studies in the Serbian population have revealed notable immunity gaps. A study conducted among university students from medical faculties in Serbia, aged 19–29 years, reported overall mumps seropositivity of 60.2%, with the lowest level (47.7%) observed in the 27–29-year age subgroup [24]. Similarly, a population-based study conducted in Belgrade, the capital city of Serbia, demonstrated overall mumps seropositivity of 85.1% across all age groups, with the lowest rate (76.1%) recorded among children aged 1–5 years (infants under 1 year of age were not included in the study) [25]. In line with the findings of the Belgrade seroepidemiological study [25], the latest available report from the European Centre for Disease Prevention and Control (ECDC) for 27 EU/EEA countries indicates that, in 2022, the most affected age group was 1–4 years [20]. In the United States (USA), the number of mumps cases declined significantly following the introduction of the mumps vaccination program in 1967. However, since 2006, increases have been observed in settings characterized by intense or frequent close contact, such as college campuses, close-knit communities, and large gatherings [19]. More specifically, between 2018 and 2023, the highest mumps incidence in the USA was reported among individuals aged 18–24 years during the 2018–2020 period, while, in 2021–2023, the highest incidence was recorded among children aged 1–4 years [26].

### 4.3. Monthly Patterns and Endemicity

Monthly data from the 1997–2024 period further illustrate the shift from sustained endemic transmission observed in the late 1990s and early 2000s to a pattern of low-level sporadic circulation in more recent years. During epidemic years—specifically in 2000 and 2012—distinct seasonal peaks were observed in the spring months. Notably, 36% of all cases in 2000 and 66% in 2012 were reported between March and May, a pattern consistent with the seasonal distribution of mumps cases in EU/EEA countries [20]. Interestingly, during a localized peak in 2009, 94% of all reported cases occurred between August and October. This small outbreak with 117 mumps cases was largely confined to a single district (Srem) in AP Vojvodina and predominantly affected previously vaccinated individuals of high school age (15–19 years) [27].

### 4.4. Vaccination Status of Cases

The analysis of cases by vaccination status across two distinct periods further supports the protective role of vaccination. During the earlier period (1997–2010), more than half of the reported cases occurred among unvaccinated individuals. In the subsequent period (2011–2024), this proportion declined; however, the percentage of cases with an unknown vaccination status increased substantially. This trend likely reflects the shifting age distribution of mumps cases and highlights documentation gaps, particularly among adolescents and adults. The observed shift toward an older age at infection—across all vaccination status categories—may also indicate waning vaccine-induced immunity, as previously described [4,6,7,8,9,12,26,28], and is consistent with findings of the aforementioned seroepidemiological studies conducted in Serbia [24,25]. These observations underscore the importance of strengthening immunization record keeping, especially in older age groups, and support the consideration of targeted strategies—such as booster doses in specific populations or high-risk settings—following an assessment of the general population’s seroepidemiological immunity profile against the mumps virus in AP Vojvodina.

### 4.5. Gender-Specific Patterns and Complications

Throughout the study period, the mumps incidence consistently exhibited a male predominance. According to data from 27 EU/EEA countries in 2022, mumps was more frequently reported among males than females across all age groups, with the exception of infants under one year of age and adults aged ≥ 20 years [20]. A similar male predominance was observed during mumps epidemics in Serbia’s neighboring countries [23,29], across various European countries [30,31,32,33,34], as well as in other regions worldwide [4,8,35,36]. The reasons for these gender discrepancies are not well understood.

Our findings indicate a slight increase in reported complications, particularly pancreatitis and orchitis, in the more recent observed period (2011–2024). Nevertheless, the vast majority of cases in both observed intervals remained clinically uncomplicated, which is consistent with the literature [1,2]. Overall, only a small proportion of complications occurred in children aged ≤ 4 years, with the exception of one case of pancreatitis reported in an infant aged < 1 year. Orchitis was identified as the most frequent mumps-related complication, which is clinically relevant given that approximately 30% of post-pubertal males with mumps orchitis may experience infertility or subfertility [37,38]. In our study, all cases of orchitis occurred among individuals aged 20–39 years, with the exception of one case in an 18 year old. Meningitis was registered in individuals aged 10–19 and 20–39 years. These findings suggest a clear age-related pattern in the occurrence of mumps complications, with orchitis and meningitis predominantly affecting adolescents and young adults. Importantly, no complications were reported among individuals who had received a single dose of the MMR vaccine, except for two cases of pancreatitis. Likewise, except for one case of orchitis, no complications were recorded among individuals vaccinated with two doses. In contrast, most complications were observed among unvaccinated individuals or those with an unknown vaccination status. These results support the protective effect of MMR vaccination—particularly two-dose immunization—against severe mumps-related complications, which is consistent with findings from previous studies [30,39,40,41,42].

### 4.6. Laboratory Confirmation and Surveillance Quality

The transition from predominantly C/E-linked case definitions to the increased use of laboratory confirmation represents a major improvement in the quality of mumps surveillance. While laboratory confirmation was limited in earlier years, it became more consistent after 2009, in accordance with the WHO recommendations [13]. This discrepancy between C/E-linked and laboratory-confirmed cases is not uncommon. Similar distributions have been previously reported. For example, according to the latest ECDC report for 2022, 42% of all reported mumps cases in EU/EEA countries were laboratory-confirmed [20]. In a study from the USA covering the period from 1 January 2018 to 31 December 2023, 47% of mumps cases were confirmed, while 53% were classified as probable [26]. In our study, the discrepancy between laboratory-confirmed and unconfirmed mumps cases was even more pronounced, particularly during the epidemic years of 2000 and 2012. Several factors may explain these differences. First, mumps surveillance in our country is primarily passive, which may contribute to a tendency toward reporting based on clinical presentation alone, without the requirement for laboratory confirmation. Second, in the early years of implementing mumps diagnostics, laboratory confirmation was only available at the Institute of Virology, Vaccines and Sera “Torlak” in Belgrade, while the diagnostic capacity of the IPHV, Novi Sad was developed at a later stage. Third, during epidemic periods, once the initial cases are laboratory-confirmed, subsequent cases are more frequently reported based on clinical criteria, particularly when there is a known epidemiological link to confirmed cases. Finally, it is well documented that the laboratory confirmation of mumps is challenging. Serological tests among vaccinated individuals must be interpreted with caution, as mumps-specific IgM antibodies are detectable in only 46–71% of RT-PCR-confirmed cases [1,2,43]. On the other hand, RT-PCR testing for mumps is most reliable when performed early in the course of illness, ideally within the first three days after symptom onset [1,2,44,45,46].

### 4.7. Public Health Implications

The findings of this study carry important public health implications. While the overall decline in mumps incidence highlights the long-term benefits of MuCV vaccination, the re-emergence of small outbreaks and the shifting age distribution of cases reveal critical gaps in population-level immunity. As previously described in detail [15], several challenges have influenced the decline in MMR vaccine coverage in our country. These include delays in the timely availability of the vaccine; increasing vaccine hesitancy due to the perceived low risks of measles, mumps, and rubella; mistrust in medical authorities; misinformation—particularly in urban areas—and persistent concerns about vaccine safety. Additionally, messages disseminated by anti-vaccination movements have further undermined public confidence in immunization. Although it is well established that the effectiveness of the Jeryl Lynn-containing MMR vaccine in preventing mumps is approximately 72% after one dose and 86% after two doses [47], maintaining high coverage with both doses remains essential to prevent future outbreaks and to mitigate the severity of the disease among those affected [1,2,5,10,18,20,26,37,38,39,40,41,42].

In line with our findings, the high proportion of mumps cases with an unknown immunization status highlights the urgent need to strengthen immunization documentation systems. The implementation of digital vaccination registries that enable lifelong tracking would significantly improve the accuracy of vaccination records and support public health responses.

Given the upward shift in the age of mumps infection and some evidence of waning immunity to the mumps virus in certain population groups in our country [24,25], and the fact that the duration of protection against mumps following MMR vaccination is estimated at 10–15 years, consideration should be given to the use of MMR booster doses in mumps outbreak settings, particularly among adolescents and young adults. Several countries, including the USA, have already introduced a third MMR dose as an outbreak control measure [48,49,50], and this approach may warrant evaluation in Serbia and similar settings. Waning of vaccine-induced immunity against mumps over time following the second dose has been identified as a contributing factor to the persistence of outbreaks in highly vaccinated populations [18,48]. A recent longitudinal study assessed the persistence of neutralizing antibodies (nAbs) against measles, mumps, and rubella over a 10-year period following the second (MMR II) and third (MMR III) doses of the MMR vaccine. The findings demonstrated that, although the nAb levels gradually declined over time, the majority of individuals remained seroprotected, especially against measles and rubella. Namely, ten years after MMR II, protective immunity was maintained in 93.7% for measles, 83.9% for rubella, and 73.4% for mumps. Similarly, 9–11 years after MMR III, 90.5% remained protected against measles, 100% against rubella, and 69.1% against mumps. The authors concluded that antibody waning was most pronounced for mumps, with a significant proportion of individuals falling below the protective thresholds over time, and suggested that waning mumps immunity may contribute to breakthrough infections in certain populations [51].

The occurrence of mumps cases in infants, particularly in the last observed year, further reinforces the importance of herd immunity. Maintaining high vaccination coverage at the community level remains the most effective means of protecting those who are too young to be vaccinated [1,2,18].

Finally, ongoing efforts to strengthen laboratory capacities and ensure consistent case confirmation are essential to improve the precision of surveillance and to support evidence-based public health decision making [13,20,43].

### 4.8. Strengths and Limitations

This study has several notable strengths. First, it presents a comprehensive, long-term analysis of mumps epidemiology in AP Vojvodina, Serbia, spanning a 47-year period (1978–2024) and encompassing various phases of immunization policy. By integrating data on incidence trends, vaccination coverage, and the demographic and clinical characteristics of cases, as well as diagnostic confirmation methods, the study provides a multifaceted perspective that is rarely reported in the regional literature. Second, the analysis was based on a mandatory, centralized communicable disease surveillance system, which ensured a high degree of completeness and consistency in data collection. Third, the inclusion of monthly case counts and age-specific analyses enabled the detection of temporal and demographic shifts in mumps transmission dynamics, particularly during epidemic periods. Finally, the stratification of cases by vaccination status allowed for a meaningful evaluation of the protective effects of one- and two-dose MMR schedules in preventing severe outcomes.

Nevertheless, several limitations should be acknowledged. First, as a retrospective observational study based on administrative surveillance data, the findings are subject to the inherent constraints of routine notification systems, including the underreporting or misclassification of cases, particularly in the earlier years of surveillance, as well as during the COVID-19 pandemic years (2020–2023), when cases may have gone unreported or patients may not have sought healthcare. In this context, we acknowledge that many mumps infections likely remained unrecognized, unclassified, unreported, or asymptomatic—an issue previously described as a major shortcoming in mumps surveillance [2,52,53,54]. Although the purpose of surveillance is not necessarily to capture every case, but rather to provide rapid insights into trends and changes in trends to support timely public health decision making [1,6,10,13,19], we acknowledge this limitation. Second, laboratory confirmation of mumps cases was limited, especially before 2009, leading to greater reliance on clinical presentation and epidemiological links for case classification. Third, data on vaccination status were incomplete for a substantial proportion of cases, particularly after 2010. Fourth, the administrative method used to estimate vaccination coverage may overestimate actual coverage, as it does not account for population movements or errors in the denominator. Lastly, potentially important confounders—including socioeconomic status, mobility patterns, and waning immunity—could not be assessed due to the absence of individual-level exposure or serological data for the entire study period.

Despite these limitations, this study provides valuable insights into the epidemiological patterns of mumps in a real-world setting over an extended period and underscores critical areas for improvement in surveillance systems, immunization program performance, and outbreak preparedness.

## 5. Conclusions

Although a noticeable decline in MMR vaccination coverage has been observed in AP Vojvodina, particularly since 2012, no major mumps outbreaks—only sporadic cases—have occurred during this period, although ongoing endemic virus transmission cannot be ruled out. In contrast, a measles outbreak and sustained endemic transmission have been recorded in the region [15,55]. This discrepancy is likely attributable to the factors discussed earlier, as well as the difference in basic reproduction numbers (R_0_) for the two diseases. Specifically, the R_0_ for measles is estimated to range between 12 and 18, whereas, for mumps, it is substantially lower, between 4 and 7 [56,57]. Consequently, the herd immunity threshold necessary to prevent measles transmission exceeds 95%, while the threshold for mumps is estimated to be approximately 75–86% [57,58]. As a result, moderate declines in vaccination coverage are more likely to precipitate measles outbreaks than mumps outbreaks. However, considering that missed vaccinations have accumulated over several years in our territory, and given the limited duration of vaccine-induced protection against mumps, it is only a matter of time before another outbreak occurs—potentially resembling those documented in 2000 and 2012 in AP Vojvodina.

Addressing declining immunization trends through targeted public health interventions remains essential for sustained mumps control in our setting. These findings underscore the urgent need to strengthen mumps prevention strategies, including the enhancement of surveillance systems (with the potential integration of active components), the achievement and maintenance of high two-dose MMR coverage, conducting a seroepidemiological study to estimate the susceptible population for mumps in AP Vojvodina, and improved outbreak preparedness. In view of the current epidemiological situation in neighboring countries, the relevance of these measures is further amplified.

## Figures and Tables

**Table 1:** Distribution of uncomplicated mumps cases and mumps-related complications by period, age, and vaccination status in AP Vojvodina, 1997–2024.

Variable		Diagnosis (*n* = 1358)			
		Uncomplicated Mumps *n* = 1326 *n* (%)	Orchitis *n* = 19 *n* (%)	Pancreatitis *n* = 5 *n* (%)	Meningitis *n* = 8 *n* (%)
Period	1997–2010	895 (67.5)	8 (42.1)	0 (-)	7 (87.5)
	2011–2024	431 (32.5)	11 (57.9)	5 (100.0)	1 (12.5)
Age group (in years)	<1	17 (1.3)	0 (-)	1 (20.0)	0 (-)
	1–4	118 (8.9)	0 (-)	0 (-)	0 (-)
	5–9	247 (18.6)	0 (-)	2 (40.0)	0 (-)
	10–19	498 (37.6)	1 (5.3)	2 (40.0)	5 (62.5)
	20–39	378 (28.5)	18 (94.7)	0 (-)	3 (37.5)
	≥40	68 (5.1)	0 (-)	0 (-)	0 (-)
Vaccination status	Two doses of MMR vaccine	182 (13.7)	1 (5.3)	0 (-)	0 (-)
	Unvaccinated	700 (52.8)	16 (84.2)	2 (40.0)	8 (100.0)
	One dose of MMR vaccine	246 (18.6)	0 (-)	2 (40.0)	0 (-)
	Unknown	198 (14.9)	2 (10.5)	1 (20.0)	0 (-)

## Data Availability

The data that support the findings of this study are available from the corresponding author upon reasonable request.

## Supplementary Materials

The following supporting information can be downloaded at: https://www.mdpi.com/article/10.3390/vaccines13080839/s1. Figure S1: Annual number of mumps cases by month and age group (<1 year = a; 1–4 years = b; 5–9 years = c; 10–19 years = d; 20–39 years = e; and ≥40 years = f) in AP Vojvodina, 1997–2024.
