# Supplementary material for: Mumps Epidemiology in the Autonomous Province of Vojvodina, Serbia: Long-Term Trends, Immunization Gaps, and Conditions Favoring Future Outbreaks
Source: Vaccines (Basel). 2025 Aug 6;13(8):839. doi: 10.3390/vaccines13080839 (PMC12390034; doi:10.3390/vaccines13080839)
Supplement: Supplementary file 1 [file vaccines-13-00839-s001.zip › vaccines-3788098-supplementary.pdf]

Figure S1. Annual number of mumps cases by months and age groups (< 1 year=a; 1-4 years=b; 5-9 years=c; 10-19 years=d; 20-39 years=e, and ≥ 40 years=f) in AP Vojvodina, 1997–2024.

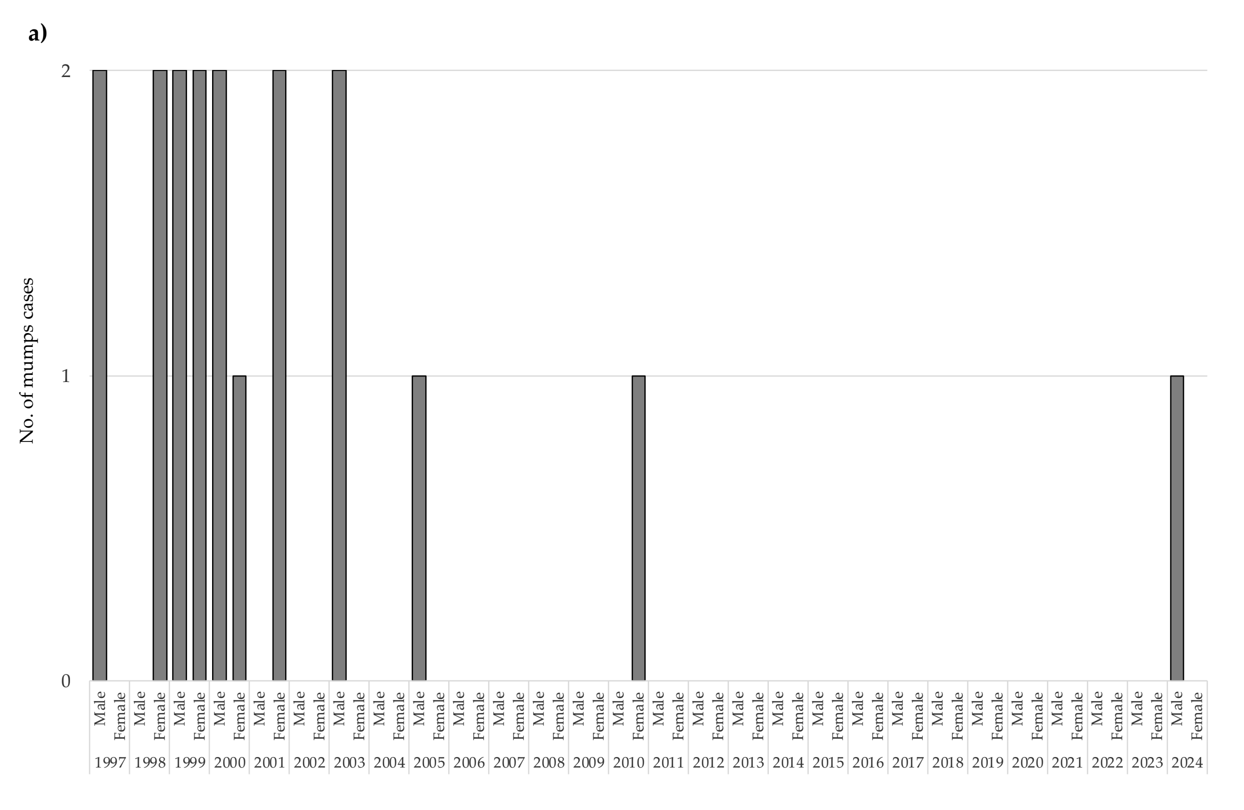

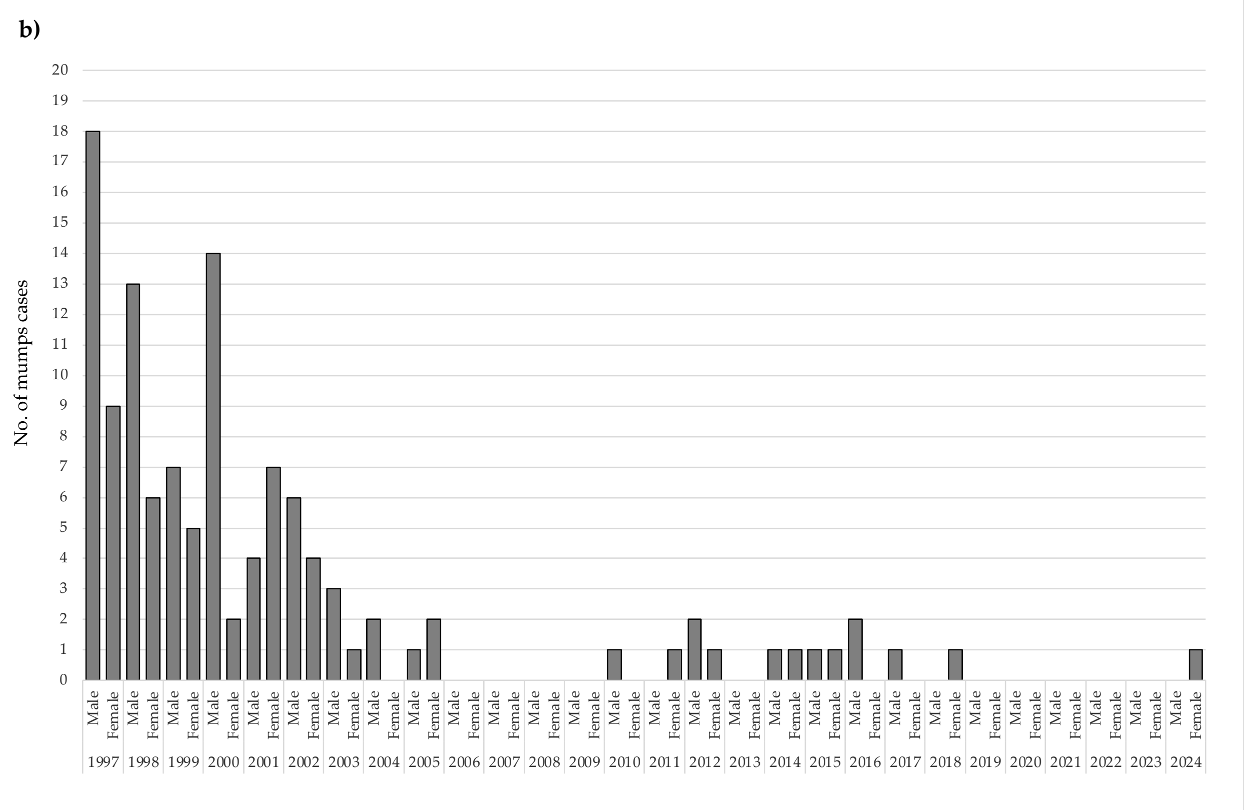

c)

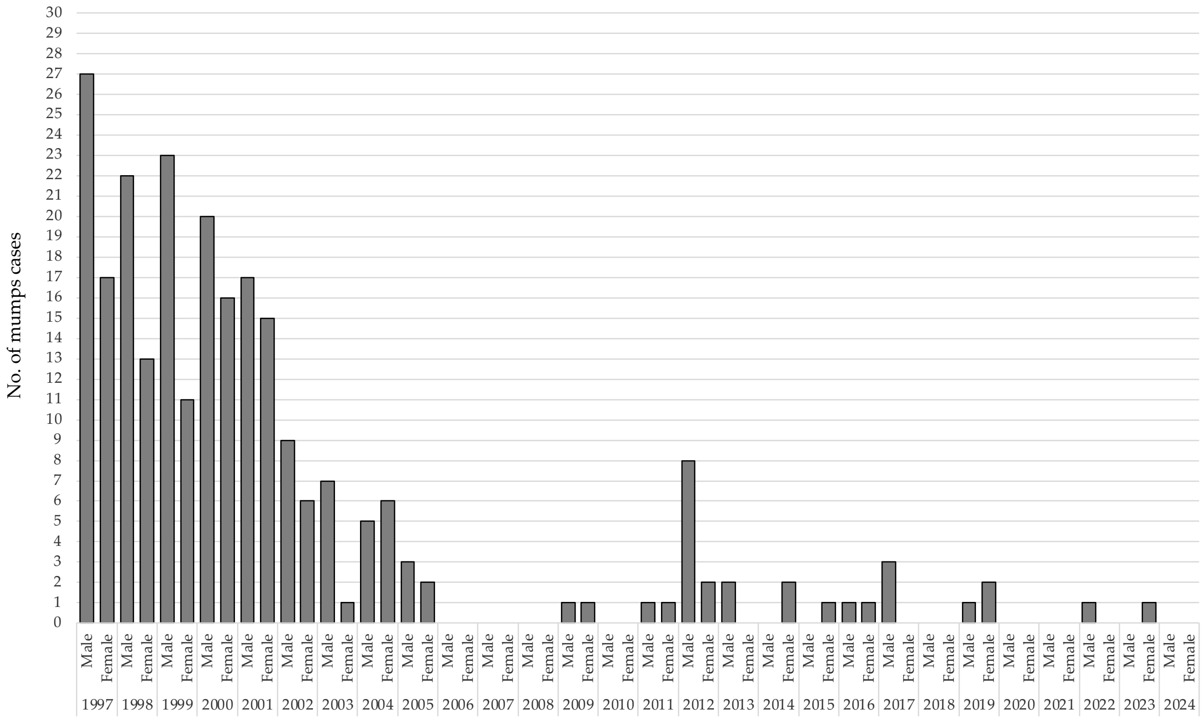
No. of mumps cases
Male
Female
1997 1998 1999 2000 2001 2002 2003 2004 2005 2006 2007 2008 2009 2010 2011 2012 2013 2014 2015 2016 2017 2018 2019 2020 2021 2022 2023 2024

d)

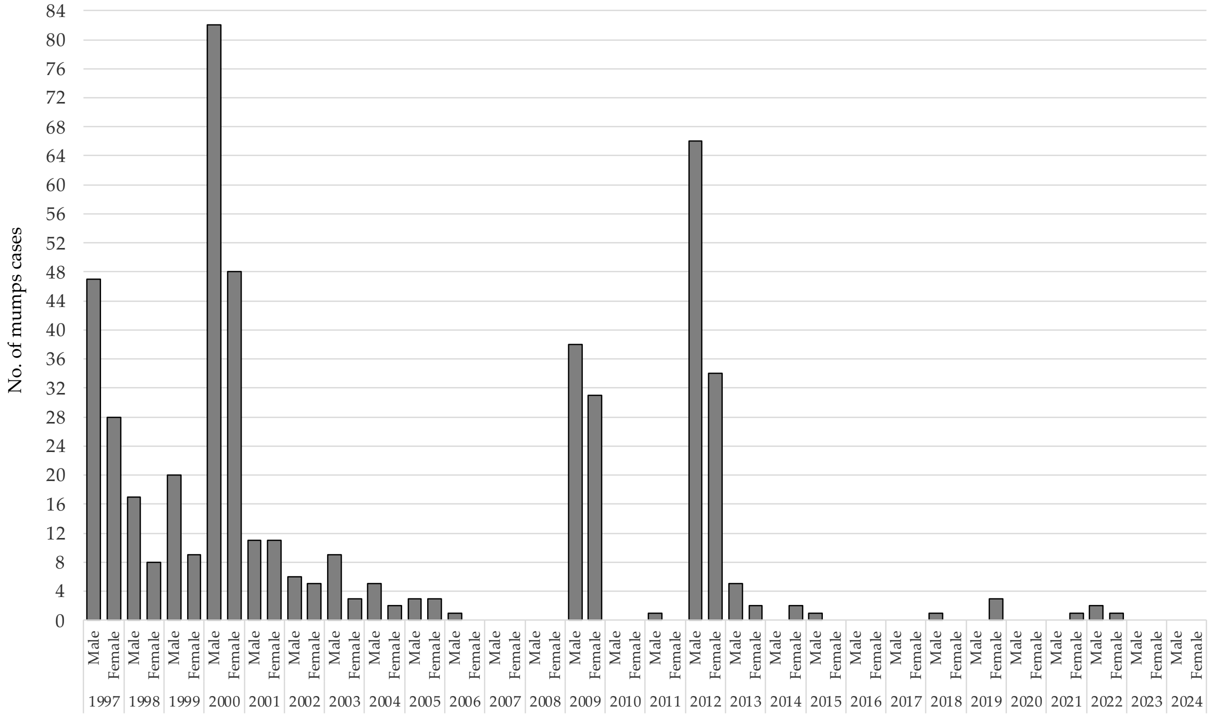
No. of mumps cases
Male
Female
1997 1998 1999 2000 2001 2002 2003 2004 2005 2006 2007 2008 2009 2010 2011 2012 2013 2014 2015 2016 2017 2018 2019 2020 2021 2022 2023 2024

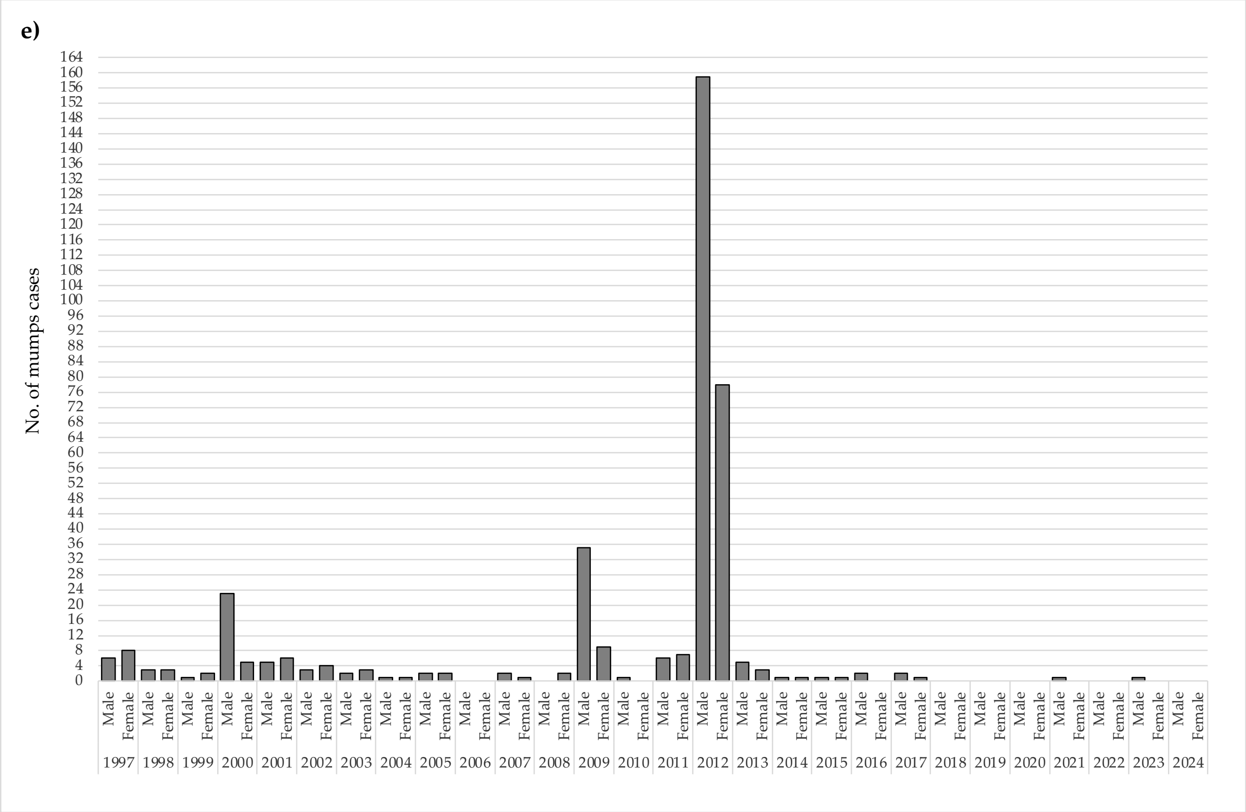
e)
No. of mumps cases
Male
Female
1997 1998 1999 2000 2001 2002 2003 2004 2005 2006 2007 2008 2009 2010 2011 2012 2013 2014 2015 2016 2017 2018 2019 2020 2021 2022 2023 2024

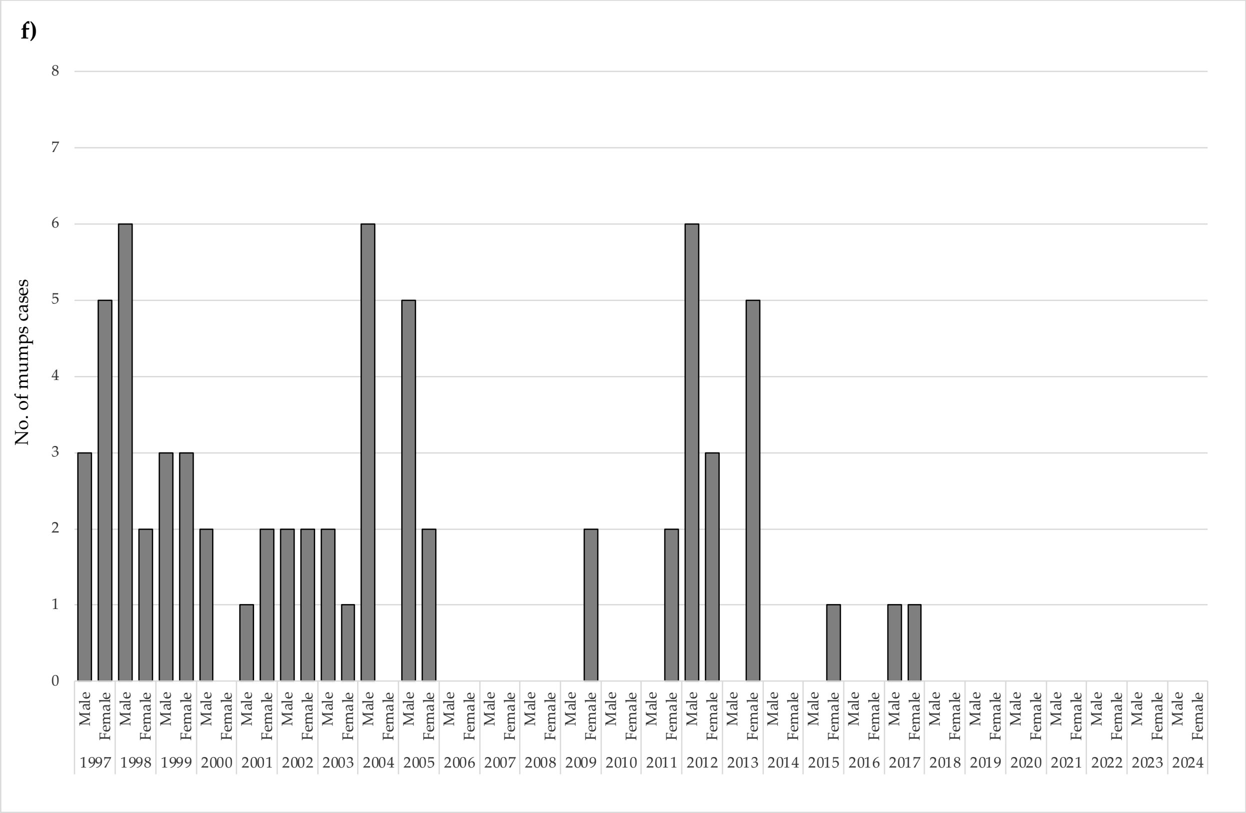
f)
No. of mumps cases
Male
Female
1997 1998 1999 2000 2001 2002 2003 2004 2005 2006 2007 2008 2009 2010 2011 2012 2013 2014 2015 2016 2017 2018 2019 2020 2021 2022 2023 2024
